# The role of budget impact and its relationship with cost-effectiveness in reimbursement decisions on health technologies in the Netherlands

**DOI:** 10.1007/s10198-024-01673-3

**Published:** 2024-02-27

**Authors:** Vivian Reckers-Droog, Joost Enzing, Werner Brouwer

**Affiliations:** 1 Erasmus School of Health Policy & Management, Erasmus University Rotterdam, P.O. Box 1738, 3000 DR Rotterdam, The Netherlands; 2 National Health Care Institute, Diemen, The Netherlands

**Keywords:** Budget impact analysis, Cost-effectiveness analysis, Decision-making framework, Reimbursement decisions, A13: Relation of Economics to Social Values, D61: Allocative Efficiency • Cost–Benefit Analysis, D63: Equity, Justice, Inequality, and Other Normative Criteria and Measurement, I13: Health Insurance, Public and Private, I18: Government Policy • Regulation • Public Health

## Abstract

Health authorities using cost-effectiveness analysis (CEA) for informing reimbursement decisions on health technologies increasingly require economic evaluations encompassing both CEA and budget impact analysis (BIA). Good Research Practices advocate that the economic and clinical assumptions underlying these analyses are aligned and consistently applied. Nonetheless, CEAs and BIAs often are stand-alone analyses used in different stages of the decision-making process. This article used policy reports and Ministerial correspondence to discuss and elucidate the role of budget impact and its relationship with cost-effectiveness in reimbursement decisions in the Netherlands. The results indicate that CEAs and BIAs are both considered important for informing these decisions. While the requirements regarding CEAs—and application of the associated decision rule—are consistent across the different stages, the same does not hold for BIAs. Importantly, the definition of and evidence on budget impact differs between stages. Some important aspects (e.g. substitution and saving effects) typically are considered in the assessment and appraisal stages but are seemingly not considered in price negotiations and the final reimbursement decision. Further research is warranted to better understand why BIAs are not aligned with CEAs (e.g. in terms of underlying assumptions), vary in form and importance between stages, and do not have a clear relationship with the results of CEAs in the decision-making framework. Improving the understanding of the circumstances under which decision-makers attach a relatively larger or smaller weight to (different aspects of) budget impact may contribute to increasing the transparency, consistency, and optimality of reimbursement decisions in the Netherlands.

## Introduction

Economic evaluations of new health technologies are increasingly used to inform reimbursement decisions in healthcare. These economic evaluations often take the form of cost-effectiveness analyses (CEA; broadly including cost–utility analysis), in which the incremental costs and benefits of the new health technology are estimated relative to a relevant comparator, e.g. “standard care” [1]. The incremental cost-effectiveness ratio (ICER) of a new health technology that results from a CEA is then evaluated against some monetary threshold (commonly expressed in terms of the opportunity costs of the health technology’s reimbursement or the societal value of a unit of benefit) to assess the value for money of its reimbursement [2, 3]. CEAs can be performed from a healthcare perspective, typically with the objective to maximize population health from a healthcare budget that is (often assumed to be) fixed, or from a broader societal perspective, typically with the objective to maximize social welfare from a healthcare budget that is (often assumed to be) more flexible [2].

In countries that apply CEAs for informing reimbursement decisions in healthcare, guidelines, and requirements regarding CEA and—more or less—well-defined decision-making frameworks are often in place [4, 5]. Health authorities in these countries, however, do not only use information on the cost-effectiveness of a new health technology when making an informed decision on its reimbursement. Increasingly, they also require evidence on other patient, disease (e.g. relating to severity), and technology characteristics, as well as a more comprehensive economic evaluation that includes a CEA and a complementary analysis of the budget impact of the technology’s reimbursement [4, 5]. Moreover, evidence suggests that the latter analysis may be an important predictor of the outcome of a reimbursement decision, also after controlling for a technology’s cost-effectiveness [6, 7]. The aim of a budget impact analysis (BIA) is to assess the financial consequences of the reimbursement from the perspective of the budget holder and the diffusion of the new technology within a healthcare system, relative to other health technologies currently used for treating the disease or condition for which the technology is indicated [4, 5]. It has been suggested to incorporate budget impact within a decision framework based on CEA by varying the monetary thresholds used to evaluate the ICER of a health technology [8]. In particular, a lower (more stringent) threshold may be applied to evaluate the ICERs of a technology with a larger potential budget impact [8].

In recent years, the focus has been on developing guidelines and formalizing requirements regarding the methodological aspects of a BIA, as well as the alignment between BIA and existing CEA methodologies [4]. Good Research Practices now advocate that the CEA and BIA of a health technology should be consistent in terms of the clinical and economic assumptions underlying the two types of analyses [5, 9]. Nevertheless, CEAs and BIAs seemingly are stand-alone analyses used separately and differently, also in terms of the underlying assumptions and the relevant stages of the decision-making process [4, 5, 9]. Less focus has been directed at clearly defining the conceptual relationship between CEA and BIA [6, 7], and developing guidance on when and why CEA and BIA should be applied separately or jointly at different stages of the decision-making process.

A coherent argument framework for using BIA, not so much in addition to but rather *in relation to* CEA, for informing reimbursement decisions has not yet been established in the Netherlands. This needs not but may be a problem when reimbursement of a cost-effective (evaluated against a relevant threshold) health technology imposes large demands on the available budget, e.g. when an expensive technology is indicated for a substantially large patient group [6]. This may also be a problem when reimbursement of a cost-ineffective health technology would impose only marginal demands on the available budget, e.g. when a technology is indicated for a relatively small patient group such as an orphan drug. While the role of cost-effectiveness in the decision-making framework in the Netherlands is clear, the role of budget impact is less transparent. It remains unclear in which stages of the decision-making process, and to what end, information on the budget impact is considered relevant and assessed by decision-makers. Furthermore, it remains unclear whether and how information on budget impact and cost-effectiveness of a new health technology are reconciled and integrated into the decision-making framework. Despite this lack of clarity, it is apparent that information on cost-effectiveness *and* budget impact currently both impact the outcomes of reimbursement decisions in the Netherlands [10].

This article aims to discuss and elucidate the role of budget impact and its relationship with the cost-effectiveness criterion in reimbursement decisions in the Netherlands, using policy reports and Ministerial correspondence on this topic. The article is structured as follows: First, we discuss the objectives of CEAs and BIAs, and how their outcomes are used for informing reimbursement decisions in healthcare. Then, we discuss the role of CEAs and BIAs in the different stages of the decision-making process, the policy implications, and evidence gaps in using the results of these analyses alongside each other to inform reimbursement decisions in the Netherlands. We conclude the article by discussing key areas for further research with the objective to increase transparency and consistency in reimbursement decisions within the Dutch healthcare system. To illustrate how CEA and BIA are currently applied in the Netherlands and structure this paper, we use the decision-making framework employed by the National Health Care Institute (ZIN) and Ministry of Health, Welfare and Sport (VWS). It should be noted that within this framework the cost-effectiveness of a health technology is directly related to the disease severity of the patients for whom the technology is indicated [11, 12]. Although this is illustrative of the decision-making frameworks in an increasing number of countries [13, 14], the issues addressed here also have relevance for countries using other decision frameworks. Also it should be noted that the decision-making framework in the Netherlands is currently mainly applied for reimbursement decisions on pharmaceuticals, although ZIN and the Ministry of VWS are planning to extend the framework to other types of health technologies [15].

## The objectives and outcomes of cost-effectiveness and budget impact analyses

Two important objectives of decision-makers in healthcare are (i) to ensure that reimbursement decisions result in value for money and (ii) to stay within the available budget, or—put more broadly—to ensure expenditures are sustainable over time [16]. CEAs provide insight into the value for money of reimbursing a new health technology in comparison to standard care and may aid decision-makers in meeting the former objective [16]. BIAs, in turn, provide insight into the expected change in healthcare expenditure after reimbursing the technology (or, in case of disinvestment, after no longer reimbursing it) and, as such, may aid decision-makers in meeting the latter objective [16]. While the outcomes of CEAs provide a single, general estimate that may be similarly assessed and used by different decision-makers at different stages in the decision-making process, the outcomes and use of BIAs may be more diverse and they may be based on other assumptions at different stages or when compared to a CEA [5]. Indeed, BIAs (ideally) reflect scenarios that provide insight into the expected change in (healthcare) expenditure—typically within one to five years—after reimbursing the new health technology and are typically tailored to *the specific needs, aims, and requirements of decision-makers* at a specific stage in the decision-making process [5, 9, 17].

CEAs and BIAs are often both used to inform the same reimbursement decisions in healthcare and, as such, they can be considered complementary [16]. Although it has been recognized that CEA and BIA are not fully independent in informing such decisions [5], more formal combinations of the two types of analyses are uncommon in decision-making frameworks. Moreover, the two types of analyses may also be seen as contributing to assisting decision-makers in meeting different objectives and can be performed based on different types of (economic) evidence [16]. Furthermore, they may be used to inform decisions made by different decision-makers at different stages of the decision-making process [16]. The differences between CEA and BIA can be substantial. They may vary in terms of the underlying assumptions and the stages of decision-making they inform. Such differences, as well as a lack of transparency regarding the relationship between and relative weight of the outcomes of CEA and BIA, may ultimately also have consequences for the transparency and consistency of reimbursement decisions in healthcare.

## The role of cost-effectiveness and budget impact analyses in the Netherlands

In the Netherlands, decision-makers at the National Health Care Institute (ZIN) advise those at the Ministry of Health, Welfare and Sport (VWS) regarding the reimbursement of new health technologies, i.e. on the desirability of their inclusion in the basic benefits package of the health insurance scheme that is mandatory to take out for all adult (18 + years) inhabitants. ZIN’s advice is based on the assessment and appraisal of evidence regarding the necessity (operationalized in terms of disease severity [12]), effectiveness, cost-effectiveness, and feasibility of (reimbursing) a new health technology—usually for a single medical indication [18]. Of these four decision criteria, ‘effectiveness’ is currently the only statutory criterion in the Netherlands. Currently, the Minister of Health is considering legislating all four criteria to curb the growth in expenditures, not only for pharmaceuticals—including those used for inpatient care—but also for all types of health technologies [15, 19]. In principle, health technologies are currently reimbursed from public funding when they meet the effectiveness criterion, which is mostly not formally a priori evaluated but based on acceptance by the relevant professionals (referred to as the ‘open system’) [20, 21]. However, pharmaceuticals used in outpatient care and specialty pharmaceuticals used for inpatient care are only reimbursed when they are explicitly placed on a positive list, which requires them to a priori provide evidence on also meeting the necessity, cost-effectiveness, and feasibility criteria (referred to as the ‘closed system’) [18, 21].

The necessity, effectiveness, and cost-effectiveness criteria are integrated into a transparent and coherent decision-making framework, in which the incremental cost-effectiveness ratio (ICER) of a health technology is evaluated against monetary reference values that are directly related to (i.e. equity-adjusted based on) the disease severity experienced by the patients [11, 12, 18]. The final criterion is (now treated as) complementary and directed at answering the question whether it is “feasible and sustainable” to reimburse a particular health technology from the basic benefits package [18]. To provide an answer to this question, decision-makers at ZIN assess evidence on several implementation aspects of the health technology, e.g. support for the implementation, ethical and legal aspects of the implementation, requirements on the organization of care, and the budget impact of reimbursement [18]. The metaphor of a funnel has often been used, describing the decision-making framework as a hierarchical model in which health technologies successively need to pass all criteria to be reimbursed from the basic benefits package [22]. However, ZIN now considers the criteria simultaneously, meaning that they need to be considered in combination in reaching a decision. For the first three criteria, this joint consideration is now formalized in the decision-making framework (with higher ICER thresholds for treatments of more severe diseases), but this is not the case for the criterion of feasibility (also containing budget impact).

**Fig. 1:**
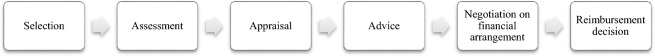
Graphic representation of the reimbursement decision-making process

The four criteria may in principle all be relevant at any stage of the decision-making process [23]. Figure 1 presents a graphic representation of the reimbursement decision-making process in the Netherlands (adapted from [21]).

This process is depicted as linear, encompassing six stages that start with the selection of (new) health technologies for evaluation—predominantly pharmaceuticals but increasingly also other types of health technologies [18]. Following their selection, the available evidence on the necessity, effectiveness, cost-effectiveness, and feasibility of these technologies is assessed and appraised [21]. Based on the results of these two stages, an advice on their reimbursement, along with supporting evidence is compiled in a report that is presented to the chair of the Board of Directors of ZIN. The chair then formulates the final advice, which is offered to the MoH. Depending on ZIN’s advice, the MoH may initiate negotiations on the establishment of a financial arrangement with manufacturers (effectuated by the Bureau Financial Arrangements Pharmaceuticals office within the Ministry of VWS) before making the final decision on reimbursement of the health technology [21].

## The role of budget impact in the different stages of the decision-making process

From what is outlined above, it may be evident that different decision-makers are involved at different stages of the full decision-making process, each with their own informational needs, responsibilities, and interests regarding the budget impact of reimbursing a health technology. The subsequent sections describe the current role of budget impact in each stage of the decision-making process to provide insight into these differences.

### Stage 1: Selection of health technologies

It is not considered “feasible or desirable” to perform a full and systematic assessment on all four decision criteria for all new health technologies that enter the market or may already be reimbursed within the healthcare system in the Netherlands [18]. Nonetheless, the conditions for such an assessment have recently been tightened in the Netherlands to reduce the risk of reimbursing especially outpatient and inpatient pharmaceuticals that are not cost effective, and hence may offer insufficient value for money [24]. New technologies for which a full and systematic assessment and appraisal of evidence on the four decision criteria is required are selected by ZIN based on an assessment of the maximum potential risk that may be associated with their reimbursement [24, 25]. This risk may include that associated with uncertainty about the effectiveness of a technology, inappropriate use of a technology in clinical practice, and publicly financing a technology [24]. The assessment of the risks associated with reimbursement of pharmaceuticals (that are expected to enter the market within the next two years) is performed by members of the Horizonscan team of ZIN in collaboration with eight disease-domain specific working groups comprising medical specialists, (hospital) pharmacists, and representatives of health insurers and patients [23]. The core task of the working groups is to validate, supplement, and rectify the relevant evidence compiled from “various national and international sources” by members of the Horizonscan team [26]—who are ultimately responsible for the content of the scan report [27].

Until July 2023, reimbursement of an inpatient pharmaceutical was considered a financial risk in case the budget impact was more than €10 million per year and the treatment costs per patient were more than €50,000 per year, or if the budget impact was more than €40 million per year [25]. If either cut-off value was exceeded, a full and systematic assessment was indicated for the pharmaceutical. As of 1 July 2023, the latter was tightened to an expected gross expenditure on the pharmaceutical of more than €20 million per year [24]. For outpatient pharmaceuticals, the criterion for requiring a full economic evaluation was having a budget impact of more than €10 million per year within the first three years of reimbursement [25]. Currently, a full assessment is indicated when the expected gross expenditure on the pharmaceutical is between €1 million and €10 million per year *and* the treatment costs per patient are more than €50,000 per year, or when the expected gross expenditure on the pharmaceutical is €10 million per year or more [24].

Given the changes to these regulations, the financial thresholds for requiring a full and systematic assessment have been lowered and the differences between inpatient and outpatient pharmaceuticals have been reduced. Furthermore, the financial risk of reimbursement is now assessed based on the expected gross expenditure *on* a pharmaceutical rather than the net budget impact *of* the pharmaceutical. This implies that any substitutions or savings resulting from reimbursing a pharmaceutical are no longer directly considered in the risk assessment. Consequently, the financial risk of reimbursement may be overestimated increasing the chances of a pharmaceutical being placed in the ‘lock’ (i.e. not being reimbursed) before a final decision is made based on a full assessment [21]. This (also referred to as ‘making the open system more closed’ [18]) may aid in reducing the risk of ‘gaming the system’ by manufacturers bypassing the assessment and appraisal of evidence in stages 2 and 3 of the decision-making process (e.g. by presenting an expected gross expenditure just below €10 million euros per year) and expediting the reimbursement advice and, potentially, also the decision. Nonetheless, it currently remains unclear on what (formal) grounds the new cut-off values for a full and systematic assessment have been determined, how the expected gross expenditure on a pharmaceutical is exactly calculated, whether any financial consequences relating to other types of risks (e.g. associated with inappropriate care of the pharmaceutical) are incorporated in estimations of expected gross expenditures, and, if so, how they are aggregated and weighted. Furthermore, it remains unclear whether (and on what grounds) these cut-off values will be applied in the future to other types of technologies. Moreover, the approach chosen adopts a narrow perspective, considering that only expenditures on pharmaceuticals are considered. This contrasts with the fact that arguably a healthcare perspective should be applied from the perspective of the budget holder to see the full impact on healthcare expenditures, and with the fact that a broader societal perspective is applied in CEAs performed in subsequent stages, which also look at broader societal costs. The difference between a healthcare and societal perspective in this context may also relate to the respective goals of performing a CEA and a BIA.

Although the differences in criteria for when and how to evaluate outpatient and inpatient pharmaceuticals have been reduced [25], differences do remain (which may also apply to other types of technologies). Indeed, substitution and saving effects (within the healthcare system) are considered for some pharmaceuticals, but not for others. The underlying reasons for such differences are not explicated. Nonetheless, considerations such as the higher likelihood that inpatient pharmaceuticals are considered ‘expensive’, partly because they are used for treatment of a smaller group of patients, and hence that their reimbursement may be assessed as being riskier may play an implicit role. In turn, gross expenditure on outpatient pharmaceuticals may be more likely to be greater, because they are often used for treatment of larger groups of patients. Whether such differences between inpatient and outpatient pharmaceuticals, also in terms of consequences, are considered fair, optimal, or desirable by the different stakeholders, including pharmaceutical companies, patients, and members of the public, remains unclear [21]. The same may hold for members of the Horizonscan team at ZIN (e.g. because this may hamper the consistency and transparency of decision-making). Insight into their views on this differentiation is currently lacking.

### Stage 2: Assessment of evidence

Pharmaceuticals enter the assessment stage of the decision-making process after being selected for a full and systematic assessment of evidence on their necessity, effectiveness, cost-effectiveness, and feasibility—usually for a single medical indication. At this stage, manufacturers are required to submit a reimbursement dossier to ZIN that meets evidence requirements that depend on whether the pharmaceutical is substitutable for other pharmaceuticals [25]. In case a pharmaceutical is not considered a therapeutic substitute of other pharmaceuticals, the results of both a CEA—which results are related directly to the disease severity of patients [11, 12]—and a BIA needs to be submitted according to standardized formats [28, 29]. In case a pharmaceutical is a therapeutic substitute of other pharmaceuticals, only the results of a BIA need to be submitted to ZIN [30].

As highlighted in the introduction to the most recent BIA format (published in 2020), a BIA provides “an estimation of the financial impact on the pharmacy/hospital budget when the new pharmaceutical is included in the basic benefits package of the mandatory health insurance in the Netherlands” and includes information on substitution and saving effects [29]. This indicates that BIAs includes more and other information than the estimation of gross expenditures in the first stage of the decision-making process. The format does imply that BIAs are performed from a narrow perspective focusing only on pharmaceutical expenditures rather than from a healthcare or societal perspective, suggesting that the Good Research Practices to align the economic and clinical assumptions underlying CEAs and BIAs are not (yet) followed [5]. It remains unclear from the available policy reports on BIAs whether this difference in perspective is deliberate or whether there are plans at ZIN to reconsider the alignment between the CEA and BIA formats. For example, by performing BIAs from a more comprehensive healthcare or even a societal perspective, and hence including expenditures that fall more broadly within or even outside the healthcare system in the Netherlands. The former would be aligned with the perspective commonly applied in BIA (i.e. that of the budget holder) and the latter would be aligned with the perspective applied in CEAs. It should be noted that this also requires a clear distinction between costs and (financial) expenditures. It furthermore remains unclear whether and, if so, how Pharmacoeconomic Advisors at ZIN (i.e. who are responsible for compiling and assessing the available evidence) and members of the independent Scientific Advisory Board (WAR) (i.e. who advice Pharmacoeconomic Advisors in this stage of the decision-making process) assess this difference in perspective [18], and what their expert views on this are.

Manufacturers and other stakeholders can provide substantive comments on the assessment report that is drafted and ultimately finalized and presented to the chair of the Board of Directors by Pharmacoeconomic Advisors at ZIN [25]. In most cases, the chair decides to base their advice to the MoH on the assessment report and WAR advice [25]. Then, the appraisal of the available evidence (stage 3 of the decision-making process) will be bypassed. In some cases, however, the chair decides to seek advice from the members of the independent Insured Package Advisory Committee (ACP) on the reimbursement decision. Then, a pharmaceutical will enter stage 3 of the decision-making process. It remains unclear from the available policy reports which role the Pharmacoeconomic Advisors have in this decision and on which criteria they or the chair base the decision to enter into stage 3. Furthermore, it remains unclear what—if any—role the budget impact of reimbursement has in this decision. Based on the available policy reports, one might conclude that pharmaceuticals with a favourable ICER and a (relatively) low budget impact are typically not appraised by the ACP, and hence do not enter stage 3 of the decision-making process. However, the latter conclusion is not substantiated by a formal decision rule, as is the case for assessment [11].

### Stage 3: Appraisal of evidence

In this stage of the decision-making process, the ACP appraises evidence on the necessity, effectiveness, and cost-effectiveness of a pharmaceutical and the feasibility of its reimbursement [31]. Specifically, the ACP appraises whether the consequences of reimbursement can be considered socially desirable based on the principles of justice and solidarity by weighting the interests of patients—for whom the pharmaceutical under appraisal is indicated as well as other patients for whom other pharmaceuticals or types of health technologies are indicated—and the general population [18]. Hence, this committee may, for example, appraise the potential impact of reimbursing the pharmaceutical in terms of crowding out other (types of) health technologies [18]. This suggests that the cost-effectiveness, as well as the budget impact of a pharmaceutical, may be considered relevant in the appraisal and therefore in the reimbursement advice drafted by the ACP. Nonetheless, what holds for the Pharmacoeconomic Advisors and WAR in stage 2 also holds for the ACP in stage 3; the available policy reports do not fully clarify what role budget impact has in this stage and how the budget impact of a pharmaceutical is weighted against its cost-effectiveness. It furthermore remains unclear what—if any—role the number of patients for whom the pharmaceutical is used has in this stage [17].

The relative influence of budget impact on the (hypothetical) reimbursement advice on a pharmaceutical has recently been examined in a discrete choice experiment administered among Pharmacoeconomic Advisors at ZIN, and members of the WAR and ACP (*n* = 58) [32]. In this study, budget impact was operationalised as the additional medical cost (i.e. 10, 50, or 100 million euros) per year spent on the pharmaceutical and its influence on the advice was compared to the influence of its cost-effectiveness, the disease severity of patients, and their health gain, as well as the profit margin of the manufacturer of the pharmaceutical [32]. The results of this study indicate that a higher budget impact negatively influences the likelihood of a positive reimbursement advice [32]. It should, however, be noted that the influence of budget impact on the advice was smaller than that of other criteria [32]. The results of this study furthermore indicate that the negative influence of a higher ICER and profit margin on a reimbursement advice may be greater in cases where also the budget impact of reimbursement is higher [32]. The latter interaction effect (also see [32]) may also be relevant in relation to the appraisal stage.

Currently, the ACP is in the process of drafting the Framework of Arguments for Expensive Pharmaceuticals [18]. In this framework, the committee describes how they weigh arguments concerning the uncertainty about the effectiveness and cost-effectiveness of expensive pharmaceuticals, unfavourable cost-effectiveness, and high budget impact, all of which are indeed said to play “an important role” in their reimbursement advice [18]. The Framework is scheduled for release by ZIN at the end of 2023 [18]. It currently remains unclear whether the ACP will address the weighting of evidence on budget impact and cost-effectiveness in the Framework, also in relation to the number of patients for whom a pharmaceutical is indicated [18]. Moreover, it is not yet clear whether the committee will also address the weighting of evidence that is in part compiled from a narrow perspective focusing on pharmaceutical expenditure (i.e. budget impact) and in part from a broader healthcare or societal perspective (i.e. cost-effectiveness) [18].

Manufacturers and other stakeholders (e.g. physicians and patient organizations) can provide verbal input during public meetings of the ACP or provide a written response to the preliminary report of the ACP before the reimbursement advice of the committee is finalized by the secretary of the ACP and presented to the chair of the Board of Directors at ZIN [31]. The reimbursement advice of the ACP may include the recommendation to the Minister of Health to negotiate a financial arrangement with the manufacturer, e.g. aimed at lowering the price of a pharmaceutical to a level that is considered societally acceptable [33]. This often implies a price reduction that drives the ICER below the maximum monetary reference value applied in CEAs [33]. Recently, the ACP appraised the reimbursement of atidarsagene autotemcel (Libmeldy®) and sacituzumab govitecan (Trodelvy®) [33, 34]. In their advice on atidarsagene autotemcel, the ACP reasoned that the impact of reimbursing the pharmaceutical would be limited in terms of crowding out other (types of) health technologies, due to its low budget impact [33]. However, in their advice on sacituzumab govitecan, the ACP did not provide any reasoning on the budget impact of its reimbursement [34]. Irrespective of the budget impact of these pharmaceuticals, the ACP advised the Ministry of VWS to negotiate a financial arrangement with each of the manufacturers. More specifically, the ACP advised to not reimburse the pharmaceuticals unless a price reduction of 60–90% for atidarsagene autotemcel and 75% for sacituzumab govitecan (in combination with a pay-for-performance reimbursement scheme) could be negotiated [33, 34]. This suggests that the cost-effectiveness of a pharmaceutical may weigh more heavily for the ACP than the budget impact of its reimbursement. Whether and why that would indeed be the case remains unclear from the available policy reports and may be clarified in the forthcoming Framework of Arguments for Expensive Pharmaceuticals [18].

### Stage 4: Advice on reimbursing health technologies

The chair of the Board of Directors is responsible for reviewing the assessment and appraisal reports that result from stages 2 and 3 of the decision-making process and sets forth the final reimbursement advice of ZIN in a letter to the Minister of Health [35]. Alongside the advice (e.g. to initiate negotiations on a financial arrangement with the manufacturer), this letter contains a summary of the evidence on the necessity, effectiveness, cost-effectiveness of the pharmaceutical and the feasibility (including the budget impact) of its reimbursement. The recommendation and summary of evidence are substantiated by the assessment and appraisal reports attached to the letter [35].

From the available advice reports, it is clear that the final advice of the Board of Directors is typically aligned with the advice of the Pharmacoeconomic Advisors, WAR, and ACP [35]. Nonetheless, it remains unclear when or why the chair of the Board of Directors would decide to diverge from their advice after reviewing the reports. Similarly, the chair’s letter to the Minister of Health contains a summary of evidence, sometimes emphasizing a criterion that was not apparently prominent in the deliberations of the ACP. For example, in the case of sacituzumab govitecan, this letter included information on the budget impact of its reimbursement, although its relevance was not apparent from the report on the ACP discussion [34, 36].

Following the advice of ZIN, the Ministry of VWS can decide to directly reimburse a pharmaceutical or to initiate negotiations with the manufacturer to establish a financial arrangement on its reimbursement. In case of the former, negotiations on a financial arrangement will not be initiated, and hence, stage 5 of the decision-making process will be bypassed. In case of the latter, negotiations will be opened by the Bureau Financial Arrangements Pharmaceuticals on behalf of the Minister of Health.

### Stage 5: Negotiation on financial arrangement

The Bureau Financial Arrangements Pharmaceuticals (installed in 2012) may initiate negotiations on a financial arrangement with the manufacturer of a pharmaceutical in case the financial risk associated with its reimbursement is considered too high [37]. In this stage, a ‘too high financial risk’ is defined as a(n above average) high expected gross expenditure *or* an unfavourable ICER [37]. This implies not only that budget impact and expenditures may play a large(r) role in this stage than in previous stages but also that substitution and saving effects may not be considered in the risk assessment by the Bureau. This is surprising considering that ZIN’s reimbursement advice contains evidence on the budget impact of reimbursement—comprising evidence on the expected gross expenditure and any substitution and saving effects [35]. This further seems to imply that the Bureau especially extracts information on the expected gross expenditure of a pharmaceutical from the available evidence on its budget impact. From the available policy reports, it currently remains unclear how the Bureau uses the evidence provided by ZIN on the necessity, effectiveness, cost-effectiveness of a pharmaceutical and the feasibility (including budget impact) of its reimbursement in its negotiations with a manufacturer (while this information is the basis for and starting of the negotiations).

Details on the negotiation process and financial arrangement are often classified and as such, it also remains unclear how the available evidence may inform the Bureau’s negotiation strategies and impact the financial arrangement [37, 38]. Nonetheless, the Minister of Health reports annually on the number of new and ongoing financial arrangements with manufacturers and on the revenues (in terms of savings on expenditures) of the Bureau without disclosing any confidential information [37]. The negotiation power of the Bureau varies between pharmaceuticals and is likely dependent on the market characteristics of a specific pharmaceutical [38]. These characteristics, for example, relate to the expected competition and expansion of medical indications for the pharmaceutical [38]. The relevance of these characteristics is evident from the annual reports on financial arrangements. In the most recent report (on financial arrangements made in 2022), the Minister of Health has, for example, indicated that “a pharmaceutical can often be used for multiple medical indications [and that] in a number of cases [the Bureau has] negotiated an [additional price] discount on medical indications for which the pharmaceutical was already reimbursed” [39]. Considering that ZIN usually advices the Minister of Health on reimbursement of a pharmaceutical for a single medical indication [39], this implies that the Bureau may still use the evidence on the budget impact of a pharmaceutical (including evidence on any substitution and saving effects) provided by ZIN but that additional evidence may be collected on the budget impact of reimbursement of the same pharmaceutical for other medical indications. It currently remains unclear whether the Bureau or ZIN collects such evidence and by whom and how this evidence is assessed and appraised in relation to the evidence on the budget impact and cost-effectiveness of the pharmaceutical for a single medical indication—which led the Bureau to initiate the negotiations. Moreover, while in stage 4 the emphasis appears to be on establishing cost-effectiveness, it remains unclear whether the Bureau has a similar aim in its negotiations.

After the negotiations between the Bureau and manufacturer are completed, the Bureau presents the results of the process to the Minister of Health, who considers the details of the potential financial arrangement and ultimately decides on the reimbursement of the pharmaceutical.

### Stage 6: Reimbursement decision

In the final stage of the decision-making process, the Minister of Health decides on the reimbursement of a pharmaceutical. In case the available evidence indicates that the pharmaceutical meets the necessity, effectiveness, cost-effectiveness, and feasibility criteria, or when the Bureau’s negotiations with the manufacturer have resulted in an acceptable ICER and/or budget impact, the Minister of Health will likely decide positively on reimbursement of the pharmaceutical. In the past, the Minister of Health has decided negatively on reimbursement only when, for example, a manufacturer decided to withdraw its application for reimbursement before price negotiations were initiated [e.g. in the case of betibeglogene autotemcel (Zynteglo®) [40]], when evidence was lacking on the effectiveness [e.g. in the case of entrectinib (Rozlytrek©) [40]], or when evidence was lacking on cost-effectiveness (e.g. ciltacabtagene autoleucel (Carvykti®) and brexucabtagene autoleucel [Tecartus®) [37]] of a pharmaceutical.

More recently, the Minister of Health has decided negatively on the reimbursement of the two pharmaceuticals atidarsagene autotemcel (Libmeldy®) and sacituzumab govitecan (Trodelvy®). In both cases, the Minister of Health commented on the decision by saying that the manufacturer “failed to meet the conditions” laid down in the negotiations on a financial arrangement [41, 42]. These conditions included lowering the price of the pharmaceutical to the level that was advised by ZIN, i.e. to a price that would result in a favourable ICER and would reduce crowding out other (types of) health technologies for other patients [41, 42]. The Minister of Health also suggested that the negotiations could be reopened by commenting that “the negative reimbursement decision could be reconsidered if the manufacturer was willing to reach an agreement on a lower price” [41, 42]. [41, 42].

In both cases, the number of patients annually affected by the decision was communicated to the public (i.e. 5 and 139 patients for atidarsagene autotemcel and sacituzumab govitecan, respectively). Furthermore, in the case of atidarsagene autotemcel, the expected expenditure *per patient* was communicated (i.e. 2.9 million euros) and in the case of sacituzumab govitecan, the expected gross expenditure *per year* was communicated (i.e. 9.6 million euros) [41, 42]. This indicates that the information provided on expected gross expenditure differs per case and that both total expenditures as well as cost-effectiveness may play a role in the final decision, and in the communication of the decision to the public.

## Key areas for further research

This article discussed and elucidated the role of budget impact and its relationship with the cost-effectiveness criterion in reimbursement decisions in the Netherlands. From the available policy reports and Ministerial correspondence, it becomes clear that ZIN and the Ministry of VWS indeed require evidence from a CEA and BIA to decide on the reimbursement of a health technology (when it follows the formal reimbursement decision-making process). Nonetheless, while the evidence required from a CEA and the application of the decision rule in relation to the results of a CEA are seemingly consistent between the different stages of the decision-making process in which this plays a role, the same does not hold for a BIA.

Importantly, evidence on—some aspects of—the budget impact of reimbursing a health technology is considered in each stage of the decision-making process. However, some important aspects of the budget impact, such as potential substitution and saving effects, typically are *only* considered in the assessment and appraisal stages of the decision-making process. Even then, the evidence on budget impact appraised and assessed in these stages is collected from a narrow perspective focusing only on pharmaceutical expenditures and it remains unclear how this relates to the healthcare perspective of the budget holder, and to the evidence on cost-effectiveness that is assessed and appraised from a broader societal perspective. It should be noted that the difference between CEA and BIA in perspectives used could also partly be overcome by using both a healthcare and a societal perspective in CEA, as has been proposed before. Moreover, using a societal perspective for a BIA, although potentially insightful, does not appear to fully align with the original objective of BIA, requires a clear definition of budgets and expenditures (rather than costs), and may be more complex.

Furthermore, ZIN includes the results of assessment and appraisal stages in their reimbursement advice to the Minister of Health, but it remains unclear whether and why the Bureau Financial Arrangements Pharmaceuticals extracts information on the expected gross expenditure of a pharmaceutical from the evidence provided on its budget impact. Furthermore, it remains unclear whether and how evidence on substitution and saving effects is considered the Bureau and whether and how) they, or ZIN, collect any additional evidence (e.g. on any other medical indications for which the pharmaceutical is reimbursed) to inform the Bureau’s negotiation strategies and the Minister of Health’s reimbursement decision. Moreover, the role of these aspects versus the role of cost-effectiveness in the negotiation stage (in other words, the aim of the process) is not fully clear. Further research is warranted to provide an answer to these questions.

The graphic representation of the decision-making process in Fig. 1 suggests that the sequence of the different stages is linear. Nonetheless, the policy reports and Ministerial correspondence discussed in this article indicate that decision-makers at different stages may decide to bypass some stages or take a step back in the process (e.g. when negotiations are reopened after a negative reimbursement decision is made). Indeed, the decision-making process can be non-linear, meticulous, and tailor made. Moreover, the type and comprehensiveness of evidence based on which the decision is ultimately based can vary between reimbursement cases. Further research is warranted to provide insight into the perceptions of decision-makers (as well as other stakeholders, such as patients and manufacturers) on this potential variation in evidence underlying reimbursement decisions. In addition, the weighting of evidence on (some aspects of) budget impact and cost-effectiveness (including the effectiveness of a health technology and disease severity of patients) may be different between health technologies. It is evident from the literature and available reports that evidence on cost-effectiveness consistently receives a large weight in the relevant stages of the decision-making process. However, it is not evident when, whether, or why evidence on (some aspects of) budget impact may receive a relatively larger or smaller weight in these stages. The soon to be expected Framework of Arguments for Expensive Pharmaceuticals will likely provide insight into the weighting of the ACP of evidence on budget impact in relation to evidence on cost-effectiveness, and the circumstances under which the weight of budget impact may be relatively larger or smaller. Further empirical research is warranted to provide insight into the (process and outcome of any) weighting of such evidence by decision-makers in other stages of the reimbursement process.

It should be noted that ZIN and the Ministry of VWS may have good reasons for keeping the details on the circumstances under which evidence on budget impact may receive a relatively larger or smaller weight in their decisions. Nonetheless, providing insight into these matters may contribute to increasing the transparency of the decision-making process, developing clear decision rules for the results of CEAs and BIAs, managing the reimbursement expectations of patients, manufacturers, and members of the public to whom Ministerial correspondence on decisions is addressed, and, ultimately, to increasing the societal support for and legitimacy of positive as well as negative reimbursement decisions.

## Conclusions

In conclusion, available policy reports and Ministerial correspondence suggest that evidence from CEAs and BIAs is considered important in informing reimbursement decisions in the Netherlands. While the requirements for CEAs—and the application of the decision rule in relation to the results—are seemingly consistent between the different stages of the decision-making process, the same does not hold for BIAs. Further research is warranted to better understand why BIAs are not aligned with CEAs (e.g. in terms of the underlying assumptions), vary in form and importance between stages in the decision-making process, and do not have a clear (and seemingly unstable) relationship with the results of CEAs. Improving the understanding of the circumstances under which decision-makers may attach a relatively larger or smaller weight to (different aspects of) budget impact in the different stages of the decision-making process also remains important. Ultimately, this may contribute to increasing the transparency, consistency, and optimality of reimbursement decisions in the Netherlands.
